# The impact of capsaicinoids on APP processing in Alzheimer’s disease in SH-SY5Y cells

**DOI:** 10.1038/s41598-020-66009-6

**Published:** 2020-06-08

**Authors:** Marcus O. W. Grimm, Tamara Blümel, Anna A. Lauer, Daniel Janitschke, Christoph Stahlmann, Janine Mett, Viola J. Haupenthal, Anna-Maria Miederer, Barbara A. Niemeyer, Heike S. Grimm, Tobias Hartmann

**Affiliations:** 1 Experimental Neurology, Saarland University, Homburg, Saar Germany; 2 Neurodegeneration and Neurobiology, Saarland University, Homburg, Saar Germany; 3 Biosciences Zoology/Physiology-Neurobiology, Faculty NT – Natural Science and Technology, Saarland University, Saarbrücken, Germany; 4 Molecular Biophysics, CIPMM, Saarland University, Homburg, Saar Germany; 5 Deutsches Institut für DemenzPrävention (DIDP), Saarland University, Homburg, Saar Germany

**Keywords:** Biochemistry, Molecular biology, Neuroscience, Diseases, Medical research, Molecular medicine, Neurology

## Abstract

The vanilloid capsaicin is a widely consumed spice, known for its burning and “hot” sensation through activation of TRPV1 ion-channels, but also known to decrease oxidative stress, inflammation and influence tau-pathology. Beside these positive effects, little is known about its effects on amyloid-precursor-protein (APP) processing leading to amyloid-β (Aβ), the major component of senile plaques. Treatment of neuroblastoma cells with capsaicinoids (24 hours, 10 µM) resulted in enhanced Aβ-production and reduced Aβ-degradation, leading to increased Aβ-levels. In detailed analysis of the amyloidogenic-pathway, both BACE1 gene-expression as well as protein-levels were found to be elevated, leading to increased β-secretase-activity. Additionally, γ-secretase gene-expression as well as activity was enhanced, accompanied by a shift of presenilin from non-raft to raft membrane-domains where amyloidogenic processing takes place. Furthermore, impaired Aβ-degradation in presence of capsaicinoids is dependent on the insulin-degrading-enzyme, one of the major Aβ-degrading-enzymes. Regarding Aβ-homeostasis, no differences were found between the major capsaicinoids, capsaicin and dihydrocapsaicin, and a mixture of naturally derived capsaicinoids; effects on Ca^2+^-homeostasis were ruled out. Our results show that in respect to Alzheimer’s disease, besides the known positive effects of capsaicinoids, pro-amyloidogenic properties also exist, enhancing Aβ-levels, likely restricting the potential use of capsaicinoids as therapeutic substances in Alzheimer’s disease.

## Introduction

**Figure 1:**
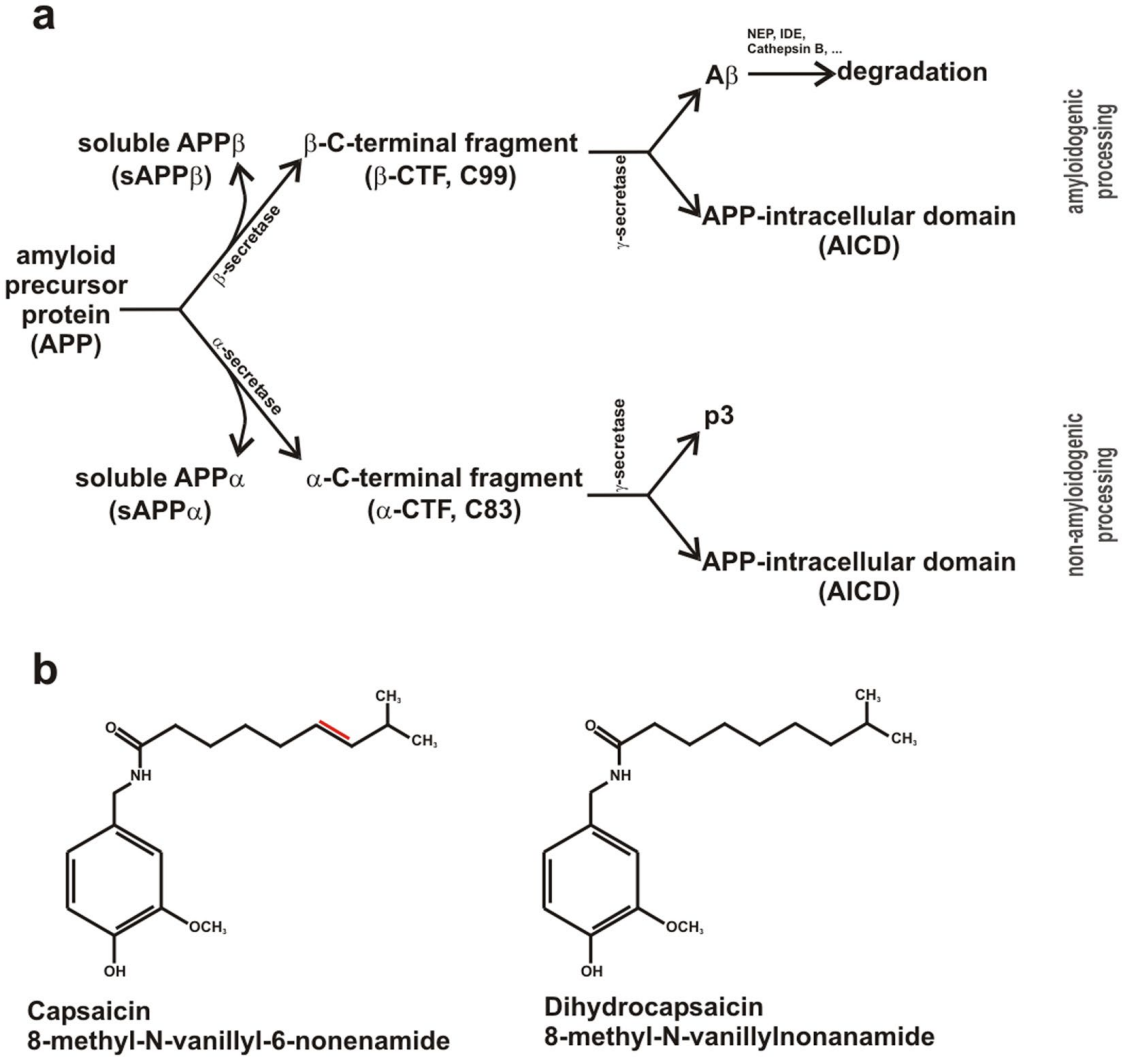
(**a**) Schematic illustration of amyloidogenic and non-amyloidogenic processing of the amyloid precursor protein (APP). (**b**) Chemical structures of capsaicin (8-methyl-N-vanillyl-6-nonenamide) and dihydrocapsaicin (8-methyl-N-vanillylnonanamide) with structural differences highlighted in red.

Alzheimer’s disease (AD) is the most common neurodegenerative disorder, characterized by degeneration of neurons in multiple brain regions, resulting in progressive memory loss and cognitive failure. Extracellular senile plaques, consisting of aggregated amyloid-β (Aβ) peptides, and intracellular neurofibrillary tangles, composed of abnormally hyperphosphorylated microtubule-associated tau-proteins, are the main histopathological hallmarks of AD^1,2^. Aβ peptides are generated by sequential proteolytic processing of the amyloid precursor protein (APP), a large type-I transmembrane protein^3^. Aβ generation involves shedding of the extracellular/luminal APP domain by β-secretase BACE1^4–6^, generating β-secreted APP (sAPPβ) and a membrane-tethered C-terminal fragment, called β-CTF or C99. β-CTF is further cleaved by γ-secretase within its transmembrane domain, releasing Aβ peptides with variable C-termini, mainly Aβ38, Aβ39, Aβ40 and Aβ42 peptides^7,8^, and the APP intracellular domain (AICD) (Fig. 1a). γ-secretase is a heterotetrameric protein complex consisting of anterior-pharynx-defective 1a or b (APH1a, APH1b), presenilin 1 or 2 (PS1, PS2), nicastrin and presenilin-enhancer 2 (PSEN2)^9,10^. The aspartylproteases PS1 and/or PS2 are the catalytically active components of the γ-secretase complex^11–13^. Besides the Aβ producing amyloidogenic pathway, APP can be processed in a non-amyloidogenic pathway by α-secretases, cleaving APP within the Aβ domain and thus preventing the release of neurotoxic Aβ peptides^14–17^. Similar to β-secretase cleavage of APP, α-secretase shedding also results in a secreted fragment, namely sAPPα. Total Aβ levels are not only dependent on the Aβ anabolism by the APP processing machinery, but also on Aβ catabolism via different mechanisms including proteolytic elimination by the main Aβ-degrading enzymes neprilysin (NEP) and insulin-degrading enzyme (IDE)^18,19^. APP and the APP-cleaving secretases are ubiquitously expressed in the human body and several physiological functions of the APP cleaving products have been described^20–26^. Furthermore, the γ-secretase complex cleaves several other substrates beside APP^27–29^. Current research focuses on modulating instead of completely inhibiting the pathways involved in Aβ generation to prevent severe side effects due to the physiological function of other secretase-mediated pathways. Several Aβ-lowering dietary components, bioactive compounds or supplements have been described, e.g. DHA, vitamin D and vitamin D analogues, polyphenols, caffeine and some phytosterols^30–36^. Recently, capsaicin, found in *Capsicum* species and spices consumed worldwide, has been proposed to be potentially beneficial in respect to tau alterations of AD in patients suffering from type 2 diabetes, known as risk factor for the development of AD^37,38^. Furthermore, it has been described that capsaicin improves cold water stress-induced AD-like pathological and cognitive impairments in rats^39^. In respect to the amyloidogenic processing and Aβ homeostasis the current literature is controversial. It has been reported that capsaicin consumption reduces blood Aβ levels in humans^40^. In contrast to the potential beneficial effects in respect to AD, a previous study described that capsaicin might promote the amyloidogenic pathway of APP processing^41^.

Capsaicin (*trans*-8-methyl-*N*-vanillyl-6-nonenamide) is the primary and naturally occurring ingredient found in *Capsicum* species, responsible for the piquancy of chili peppers. Structurally, capsaicin belongs to a group of chemicals known as vanilloids with three important regions, an aromatic head, an amide linkage and a hydrophobic tail (Fig. 1b). Beside capsaicin, more than twenty capsaicinoids were identified in *Capsicum* species^42^, varying in the length and degree of saturation in the aliphatic side chain. Capsaicin and dihydrocapsaicin (DHC) are the most prominent capsaicinoids in *Capsicum* species, accounting for 80% to 90% of the capsaicinoids^42^. Capsaicinoids possess pleiotropic pharmacological and physiological activites, including anti-inflammatory, anti-apoptotic, anti-oxidant, anti-cancer, anti-obesity, neuro-protective and analgesic functions^43–46^. Furthermore, capsaicin has been shown to have beneficial effects on cardiovascular, gastrointestinal and dermatological diseases^47–49^. Capsaicin is a highly selective agonist of the transient receptor potential channel of the vanilloid receptor family, subtype 1 (TRPV1), a Ca^2+^ permeable nonselective cation ligand-gated channel highly expressed in unmyelinated C-type sensory nerve fibers, in small-diameter sensory neurons of the dorsal root, trigeminal and vagal ganglion^50–52^ as well as in the hippocampus, striatum, hypothalamus and cerebellum of human and rat brain^53^. TRPV1 activation causes influx of extracellular calcium, resulting in elevated intracellular calcium, which is involved in a variety of physiological processes^54^. TRPV1 activation in the central nervous system contributes to basic neuronal functions including resting membrane potential, neurotransmitter release, synaptic plasticity and hippocampal memory function^55^. Also, several TRPV1-independent effects of capsaicin have been described in the past^56–59^.

As capsaicin is widely consumed as a nutritional compound or spice and the effects in respect to AD are either controversial or not investigated, we systematically examined the effects of the two major capsaicinoids, capsaicin and DHC, as well as a naturally occurring mixture on APP processing and Aβ homeostasis.

## Materials and Methods

### Chemicals and reagents

Capsaicin, dihydrocapsaicin (DHC) (analytical standards), natural occurring capsaicin and, if not stated otherwise, all other chemicals used in this study were acquired from Merck former Sigma-Aldrich (Darmstadt, Germany).

### Cell culture

Cells of the human neuroblastoma SH-SY5Y cell line were cultivated in Dulbecco’s Modified Eagle’s Medium (DMEM), containing 10% fetal calf serum (FCS, GE Healthcare Life Sciences, Chalfont St. Giles, United Kingdom) and 0.1% non-essential amino acid solution (MEM). For the African green monkey fibroblast-like cell line COS7, culture medium contained DMEM and 10% FCS. For the stably transfected cell lines SH-SY5Y/COS7 APP^695^, which overexpress the major neuronal APP isoform in humans, APP^695^, and for stably transfected SH-SY5Y C99 cell line^60^, which overexpress the β-secretase cleavage product, β-CTF, 400 µg/mL hygromycin B (PAN-Biotech, Aidenbach, Germany) were added to the media. For the murine neuroblastoma cell line (N2a), culture medium consisted of DMEM, 10% FCS, 0.1 mM MEM, 0.1 mg/mL Streptomycin, 100 U/ml Penicillin, 2 mM L-Glutamine and 1 mM sodium pyruvate. For N2a cells with IDE knock-down, promoted by SureSilencing shRNA plasmids (Qiagen, Hilden, Germany) regarding to manufacturer’s protocol and^61^, 400 µg/mL Hygromycin B were added to the media. Knock-down efficiency is shown in Supplemental Fig. S4.

### Incubations

#### Cell culture

Dependent on the following experiments, FCS in DMEM was reduced to 1%, 16 h prior incubation. FCS was reduced to elucidate the potential effect of capsaicinoids on cholesterol or other lipids being also present in FCS. Capsaicin, DHC, and natural compound were incubated in a concentration of 10 µM, Ruthenium Red was incubated at 2 µM with or without 10 µM capsaicin for 24 h (8 + 16 h), whereas controls were treated with HPLC-grade ethanol (Fisher Scientific, Schwerte, Germany) as a solvent control for the capsaicinoids and HPLC-grade water as solvent control for Ruthenium Red.

#### Cell lysates

Cells were lysed using 150 mM NaCl (VWR Chemicals, Radnor, PA, USA), 50 mM Tris/HCl pH 7.4, 2 mM EDTA (Carl Roth, Karlsruhe, Germany), 1% NP-40, 1%  Triton X-100 lysis buffer supplemented with protease inhibitor (Roche Diagnostics, Basel, Switzerland). Afterwards, the protein concentrations were determined by bicinchoninic acid assay (BCA) as described earlier^62^ and adjusted to equal protein concentrations for following experiments.

#### Cell homogenates for isolated membranes and raft fractions

Cultivated SH-SY5Y cells were washed two times with ice-cold PBS for membrane isolation, and MBS (25 mM MES, 150 mM NaCl, 0.1%  Triton X-100, pH 6.5) for raft isolation. Homogenates for membrane isolation were collected in 150 µL sucrose buffer (10 mM Tris/HCl, 200 mM sucrose, 1 mM EDTA pH 7.5). Raft-homogenates were solubilized in 500 µL MBS buffer. The solutions were then homogenized via Minilys (Peqlab, Erlangen, Germany) for 20 seconds on maximum intensity. Afterwards raft-homogenates were further homogenized via a 24 G cannula. Protein amount was adjusted to 5 mg/mL for rafts and 2 mg/mL for membrane isolation. The latter were then centrifuged at 900 rcf for 10 min at 4 °C and supernatants containing postnuclear fractions (PNFs) were collected^34^. Homogenates were stored at −80 °C.

#### Preparation of isolated membranes

PNFs were incubated with 10 µM capsaicin, natural compound or solvent control at 37 °C for 20 min while shaking. Further preparation was performed as described in^63^. For a detailed description see also supplemental information. Before measuring secretase activity, incubation was performed again as described above.

### Lipid raft preparation

Lipid raft preparation was performed as described in^32^. For a detailed description see also supplemental information.

### Western blot (WB) experiments

BACE1 and PS1 protein levels were analyzed in cell lysates, whereas for the determination of the total protein levels of the secreted proteins Aβ, sAPPα, and sAPPβ growth media were used. All samples were adjusted to the same protein amount via BCA. Antibodies and dilutions used in this study are: W02 antibody for the detection of β-CTF and total Aβ (5 µg/mL; Millipore, Billerica, MA, USA), anti-human sAPPβ (1:50; IBL America, Minneapolis, MN, USA), anti-human BACE1 B0806 (1:1000; Merck, former Sigma-Aldrich, Darmstadt, Germany), anti-human PS1 sc-7860 (Santa Cruz, Dallas, Texas, US), anti-rabbit IgG HRP Conjugate W4011 (1:5000; Promega, Mannheim, Germany) and anti-mouse P0260 (Dako, Hamburg, Germany). For detection of proteins, the enhanced chemiluminescense (ECL)-method (Perkin Elmer, Rodgau-Jügesheim, Germany) was used. The quantification was performed densitometrically with Image Gauge V3.45 software (Fujifilm, Düsseldorf, Germany).

### Immunoprecipitation

For detection of Aβ and β-CTF, immunoprecipitation of equal volumes of conditioned media or cell lysates, adjusted to the same protein amount, was performed as described in^64^. Precipitates were further used for WB experiment. For a detailed description see also supplemental information.

### Determination of total Aβ-degradation in N2a wt and N2a IDE knock down cells

N2a wt/IDE knock-down cells were reduced in FCS to 1%/DMEM 16 h before incubation. To determine the total degradation of Aβ, cells were incubated with 10 µM capsaicin or HPLC-grade ethanol as control  for 18 h. Then, incubation medium was replaced now containing not only capsaicin or ethanol, but also human synthetic Aβ40 peptide (Bachem, Bubendorf, Switzerland) (0.5 µg/mL). After 6 h, non-degraded human Aβ was detected by WB analysis using W02 antibody.

### Secretase activity assays

β- and γ-secretase activities in living SH-SY5Y wild type cells were analyzed as described in^65^. Activities of both secretases in isolated membranes and raft fractions were examined as described in^66^. For detailed descriptions of these methods see also supplemental information.

### Quantitative real-time polymerase chain reaction (RT-PCR) experiments

Gene expression analysis was performed as described in^67^. In this study the following primers were used: actin (*ACTB*): forward 5′-CTT CCT GGG CAT GGA GTC-3′, reverse 5′-AGC ACT GTG TTG GCG TAC AG-3′; anterior-pharynx-defective 1 homolog A (*APH1A*): forward 5′-GCC TCT GTG GTC TGG TTC AT-3′, reverse 5′-TCT GCC TTC TTA AGC AGC TTG T-3′; β-site APP-cleaving enzyme 1 (*BACE1*): forward 5′-GCA GGG CTA CTA CGT GGA GA-3′, reverse 5′-TAG TAG CGA TGC AGG AAG GG-3′; nicastrin (*NCSTN*): forward 5′-CTG TAC GGA ACC AGG TGG AG-3′, reverse 5′-GAG AGG CTG GGA CTG ATT TG-3′; presenilin1 (*PSEN1*): forward 5′-CTC AAT TCT GAA TGC TGC CA-3′, reverse 5′-GGC ATG GAT GAC CTT ATA GCA-3′; presenilin2 (*PSEN2*): forward 5′-GAT CAG CGT CAT CGT GGT TA-3′, reverse 5′-GGA ACA GCA GCA TCA GTG AA-3′; presenilin enhancer (*PSENEN*): forward 5′-CAT CTT CTG GTT CTT CCG AGA G-3′, reverse 5′-AGA AGA GGA AGC CCA CAG C-3′ (Eurofins MWG Operon, Eberberg, Germany). Results were normalized to actin and the 2^-(ΔΔCt)^ method was used to calculate expression changes.

### Cholesterol concentration

Cholesterol concentration was measured by using the Amplex Red Cholesterol Assay Kit (Invitrogen, Carlsbad, CA, USA) according to the manufacturer’s protocol.

### Lactate dehydrogenase (LDH) activity assay

To measure the cytotoxicity of the capsaicin incubation, Cytotoxicity Detection Kit (LDH) from Roche (Basel, Switzerland) was used according to the manufacturer’s instructions.

### Fluorescence-based Ca^2+^ imaging

To monitor intracellular calcium levels after capsaicin application, a calcium imaging setup was used as described in^68^.

SH-SY5Y APP^695^ cells were incubated on glass coverslips with 1 μM Fura 2-AM at room temperature for 25 min. A self-built perfusion chamber was used with low volume and high solution exchange rate. The external Ca^2+^ Ringer solution contained (in mM): 145 NaCl, 4 KCl, 2 MgCl2, 10 glucose, 10 Hepes and 1.2 Ca^2+^ Ringer (pH 7.4 with NaOH). After washing the cells twice, 10 μM capsaicin was added. Ca^2+^ images were recorded before, during, and after application of capsaicin. For analysis TILLVision followed by Igor software was used.

As a proof of principal, SH-SY5Y APP^695^ cells were transiently transfected with human *TRPV1* and *GFP* as negative control according to manufacturer’s guidelines 24 h before imaging.

### Thioflavin T based Aβ aggregation assay

To measure aggregation of Aβ, we modified the thioflavin T based assay described in^69^. 500 µg Aβ42 peptide (generous gift of B. Penke, Szeged, Ungary) was dissolved and incubated for 24 h in 1 mL hexafluoro-2-propanol (HFIP) at 4 °C. Monomerized peptides were then dried via SpeedVac (Savant DNA110, Thermo Fisher Scientific, Waltham, MA, USA) at room temperature. Afterwards dried peptides were resolved in 100 µl ammonium hydroxide, sonicated for 30 min on ice, and filtered via centrifugation for 90 min at 13,900 rpm and 8 °C in Amicon Ultra-0.5 mL Filters. Final Aβ concentrations were detected via UV-Vis-spectrophotometer (NanoDropTM 8000, Thermo Fischer Scientific, Waltham, MA, USA) absorbance measurement at 280 nm. 10 µM of freshly monomerized Aβ42 was incubated with 15 µM thioflavin T on a black 96 well quartz microplate (Hellma^TM^, Müllheim, Germany) in 100 µl total volume per well and in the presence of 10 µM natural compound capsaicin or ethanol as solvent control, both conditions were performed in presence of 10 µM phosphatidylcholine 16:0. Measurement was performed with an infinite M1000 Pro (Tecan, Crailsheim, Germany) at 37 °C for at least 3 days with the following wavelengths: excitation at 450 ± 5 nm and fluorescence detection at 482 ± 5 nm.

### IDE activity assay

IDE enzyme activity was analyzed as described in^70^ with minor modifications. Anti-IDE antibody ST1120 (Merck) was diluted in 1xPBS (5 µg/ml) and coated on a Nunc MaxiSorp 96-well plate for 24 hours at room temperature. After washing the plate five times with 1x PBS containing 0.5% Tween-20, blocking was performed by incubating 10% fatty acid free bovine serum albumin in 1x PBS for two hours at room temperature. Afterwards, plate was washed three times and lysates were incubated for one hour at 20 °C and 150 rpm, followed by five wash steps. After a pre-incubation with assay buffer (50 mM Tris-HCl pH 7.4, 1 M NaCl, 10 µM MgCl_2_, β-secretase inhibitor II (565749, Merck), γ-secretase inhibitor IV (565761, Merck), complete protease-inhibitor without EDTA) for 15 minutes at room temperature, the fluorogenic peptide substrate Mca-RPPGFSAFK(Dnp)-OH (10 µM) was added and fluorescent was measured using a Safire^2^ Fluorometer (Tecan, Crailsheim, Germany) at an excitation wavelength of 320 ± 10 nm, and an emission wavelength of 405 ± 10 nm.

### Statistical analysis

All quantified data represent an average of at least three independent experiments. Error bars represent standard deviation of the mean. Normal distribution was tested by Kolmogorow-Smirnow and Shapiro-Wilk tests. Statistical significance was determined by two-tailed Student’s t test for comparing two parameters or ANOVA (Games-Howell and Bonferroni *post hoc*) tests in case of multiple parameter comparison. Significance was set at *p ≤ 0.05, **p ≤ 0.01, and ***p ≤ 0.001.

## Results

### Capsaicinoids increase amyloidogenic APP processing

**Figure 2:**
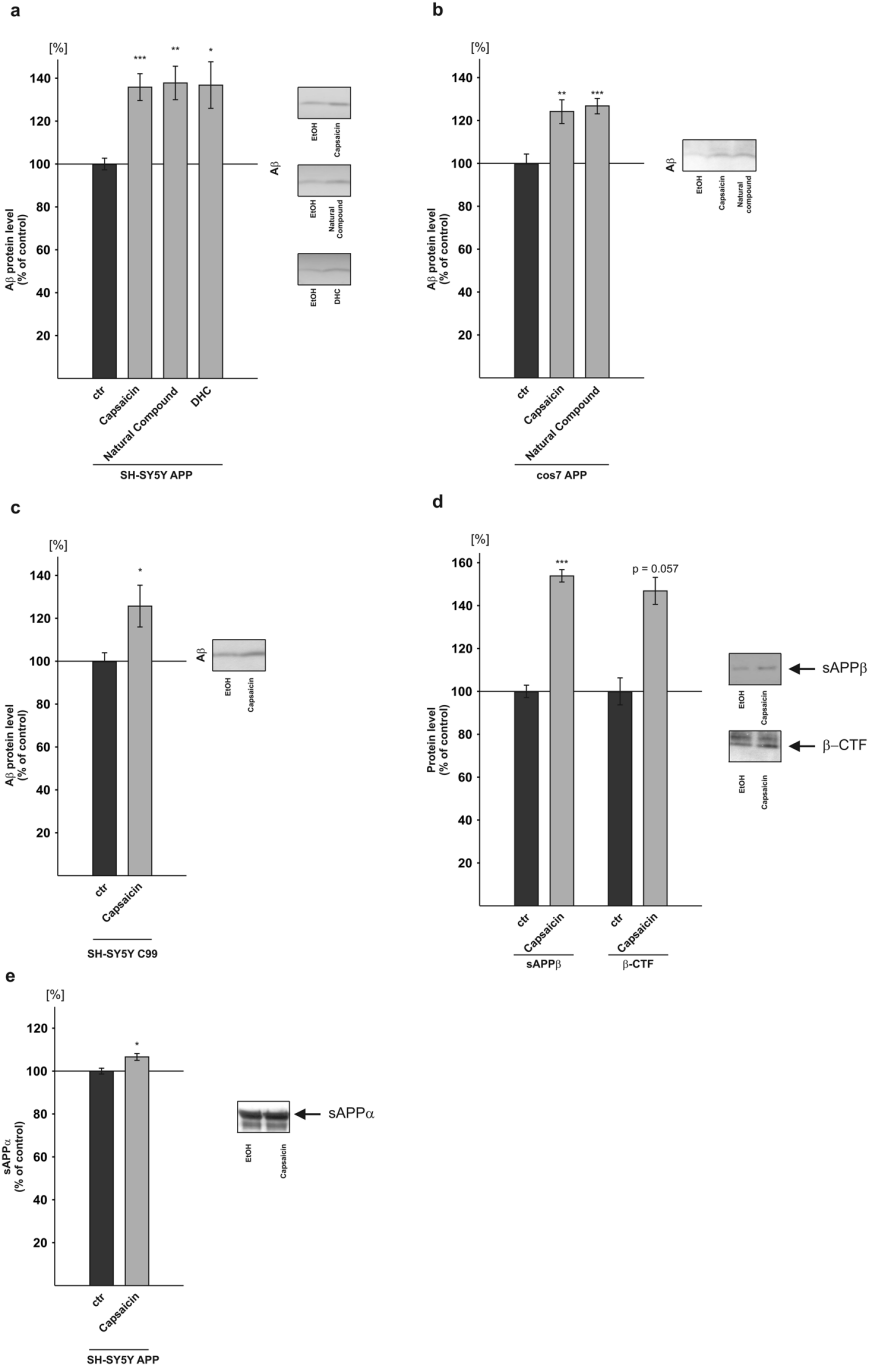
Effect of capsaicinoids on amyloidogenic processing of APP in different cell lines. (**a**) Capsaicin, natural compound and DHC were incubated on SH-SY5Y APP^695^ (n ≥ 9) cells and Aβ protein levels were analyzed in comparison to control treated cells by WB. (**b**) COS7 APP^695^ cells were treated with capsaicin and natural compound and levels of Aβ were determined (n ≥ 4). (**c**) Protein levels of Aβ were examined after incubation of capsaicin in SH-SY5Y C99 cells (n = 14). (**d**) sAPPβ and β-CTF protein levels were analyzed in SH-SY5Y APP^695^ cells after capsaicin treatment (n = 3). (**e**) Protein levels of sAPPα were analyzed in SH-SY5Y APP^695^ cells after capsaicin treatment in comparison to solvent control by WB (n = 5). Representative signals are illustrated on the right, respectively. Error bars represent the standard error of the mean. Asterisks show the statistical significance calculated by unpaired Student’s t test or ANOVA (*p ≤ 0.05; **p ≤ 0.01; ***p ≤ 0.001).

To examine the effect of capsaicinoids on APP processing, we analyzed the most predominant forms present in *Capsicum*, capsaicin (trans-8-ethyl-N-vanillyl-6-nonenamide) and dihydrocapsaicin (DHC), as well as the natural compound comprising beside capsaicin and DHC several other capsaicinoids^42^. The aliphatic side chains of capsaicin and DHC have an identical length but vary in respect to a double bond which is present in capsaicin but is lacking in DHC (Fig. 1b). As cellular systems we used three different stably transfected cell lines: human neuroblastoma SH-SY5Y cells and COS7 cells stably expressing APP^695^, the most abundant APP isoform found in neurons^71^, and SH-SY5Y cells stably expressing the truncated APP construct C99. C99 represents the C-terminal fragment of β-secretase cleaved APP and only requires cleavage by γ-secretase to release Aβ^60^. Cells were incubated for 24 hours in presence of 10 µM capsaicinoids and Aβ generation was monitored by a highly sensitive Aβ-immunoprecipitation and Western blot (WB) analysis^72^. All analyzed substances revealed a very similar increase in the Aβ levels in APP^695^ transfected SH-SY5Y cells upon capsaicin treatment: In presence of pure capsaicin, Aβ levels were significantly elevated to 136.1%; in cells incubated with the natural compound Aβ levels were significantly increased to 138.1% and DHC treated cells showed a significant elevation to 137.1% of secreted Aβ compared to cells treated with the solvent control ethanol (capsaicin: 136.1% ± 6.2%, p ≤ 0.001; natural compound: 138.1% ± 7.8%, p = 0.003; DHC: 137.1% ± 10.9%, p = 0.037) (Fig. 2a). Notably, no cytotoxicity was observed under these experimental conditions (Supplemental Fig. S1). In line with these findings capsaicinoid treated APP^695^ expressing COS7 cells, an immortalized cell line of the African green monkey, also revealed an increase in the Aβ levels. In these cells, capsaicin treatment led to a significant Aβ elevation to 124.5% and the natural compound caused a significant increase in the Aβ levels to 127.2% (capsaicin: 124.5% ± 5.6%, p = 0.002; natural compound: 127.2% ± 3.6%, p = 0.001) (Fig. 2b). The capsaicinoid induced increases in the Aβ levels were slightly reduced compared to APP^695^ transfected SH-SY5Y cells, which might be explained by the fact that in non-neuronal cells APP is primarily cleaved by α-secretase whereas neuronal cells show an elevation in β-secretase APP cleavage^73–75^. No significant differences between the single compounds were observed for both APP^695^ expressing SH-SY5Y and COS7 cells, as determined by ANOVA analysis. In order to examine the effect of capsaicin on γ-secretase processing independent of β-secretase cleavage, we analyzed SH-SY5Y cells stably transfected with β-CTF/C99. Capsaicin treated β-CTF/C99 expressing SH-SY5Y cells also showed a significant increase in the Aβ levels to 126.0% (capsaicin: 126.0% ± 9.7%, p = 0.020) (Fig. 2c), indicating that γ-secretase processing of APP is affected by capsaicinoids. However, as the effect strength for secreted Aβ levels observed in β-CTF/C99 expressing SH-SY5Y cells was slightly reduced compared to APP^695^ expressing SH-SY5Y cells, one can assume that capsaicin also affects β-secretase processing of APP. Therefore, we analyzed the APP cleavage products generated by β-secretase processing, β-secreted APP (sAPPβ) and β-CTF. In presence of capsaicin, β-CTF was increased to 147.2% in cell-lysates of APP^695^ transfected SH-SY5Y cells, though the observed increase did not reach significance (β-CTF: 147.2% ± 12.56%, p = 0.057) (Fig. 2d). However, detection of sAPPβ in the conditioned medium of capsaicin treated APP^695^ expressing SH-SY5Y cells showed a significant increase of sAPPβ to 154.2% (sAPPβ: 154.2% ± 2.9%, p ≤ 0.001) (Fig. 2d), indicating elevated β-secretase processing of APP in presence of capsaicin. Besides increasing β-secretase processing of APP, capsaicin also slightly but significantly elevated the generation of sAPPα to 113.7% (±3.2%, p = 0.017) (Fig. 2e).

### Capsaicinoids affect β-secretase activity by elevated BACE1 gene expression

**Figure 3:**
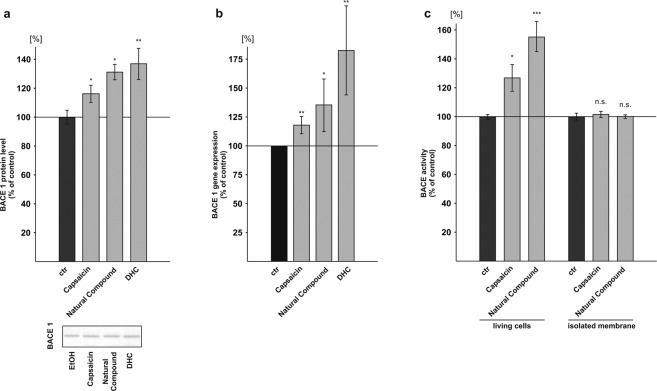
Influence of capsaicinoids on β-secretase. (**a**) Protein levels of BACE1 after incubation of SH-SY5Y APP^695^ cells with capsaicin, natural compound and DHC examined by WB (n ≥ 3). Representative signals are illustrated on the right. (**b**) *BACE1* gene expression analysis by RT-PCR after capsaicinoid treatment in SH-SY5Y cells (n ≥ 4). (**c**) β-secretase activity in living cells (n = 10) and in isolated membranes (n = 3) of capsaicin and natural compound incubated SH-SY5Y wt cells. Error bars represent the standard error of the mean. Asterisks show the statistical significance calculated by unpaired Student’s t test or ANOVA (*p ≤ 0.05; **p ≤ 0.01; ***p ≤ 0.001).

The molecular mechanism of the observed capsaicin induced elevated β-secretase cleavage of APP was further examined in neuroblastoma cells by analyzing BACE1 protein levels, gene expression and by performing β-secretase assays in living cells and purified membranes. In presence of capsaicin the BACE1 protein levels were significantly increased to 116.5% (capsaicin: 116.5% ± 5.9%, p = 0.0382). Cells incubated with the natural compound or DHC showed a significant increase in BACE1 protein levels to 131.5% and 137.3%, respectively (natural compound: 131.5% ± 5.4%, p = 0.021; DHC: 137.3% ± 10.7%, p = 0.006) (Fig. 3a). To validate these findings, we analyzed *BACE1* gene transcription by real-time (RT)-PCR analysis. All tested capsaicinoids caused a significant increase in *BACE1* mRNA levels. RT-PCR analysis of capsaicin treated cells showed a significant increase to 117.9% in *BACE1* gene transcription (capsaicin: 117.9% ± 0.06%, p = 0.005), which is in line with the observed increase in the BACE1 protein levels to 116.5%. *BACE1* mRNA levels of cells incubated with the natural compound revealed a significant increase to 135.5% and DHC elevated *BACE1* gene expression to 182.7% (natural compound: 135.5% ± 18.2%, p = 0.039; DHC: 182.7% ± 30.8%, p = 0.009) (Fig. 3b). However, the observed differences between *BACE1* expression as well as in protein levels between the single capsaicinoids did not reach significance using ANOVA analysis. To further strengthen the involvement of β-secretase processing in elevating total Aβ levels in presence of capsaicinoids, we first measured β-secretase activity in living cells. Capsaicin treated SH-SY5Y cells showed a significant increase in β-secretase activity to 127.1% and cells treated with the natural compound elevated β-secretase activity to 155.5% (capsaicin: 127.1% ± 9.3%, p = 0.042; natural compound: 155.5% ± 10.4%, p = 0.001) (Fig. 3c). Again, ANOVA analysis did not reveal a significant difference between capsaicin and the natural compound. In accordance to the observed elevation in β-secretase activity in living SH-SY5Y cells in presence of capsaicinoids, living HEK cells as well as living N2a cells treated with the natural compound also showed an increase in β-secretase activity to 124.6% and 119.4% respectively, resulting in a mean β-secretase activity enhancement for all analyzed cell lines to 133.2% (±11.3%, p = 0.042) (see Supplemental Fig. S5). In contrast to living cells, we observed no effect of capsaicin and the natural compound, when these compounds were incubated on isolated membranes, measuring a potential direct effect on β-secretase activity (Fig. 3c).

### Influence of capsaicinoids on γ-secretase activity

**Figure 4:**
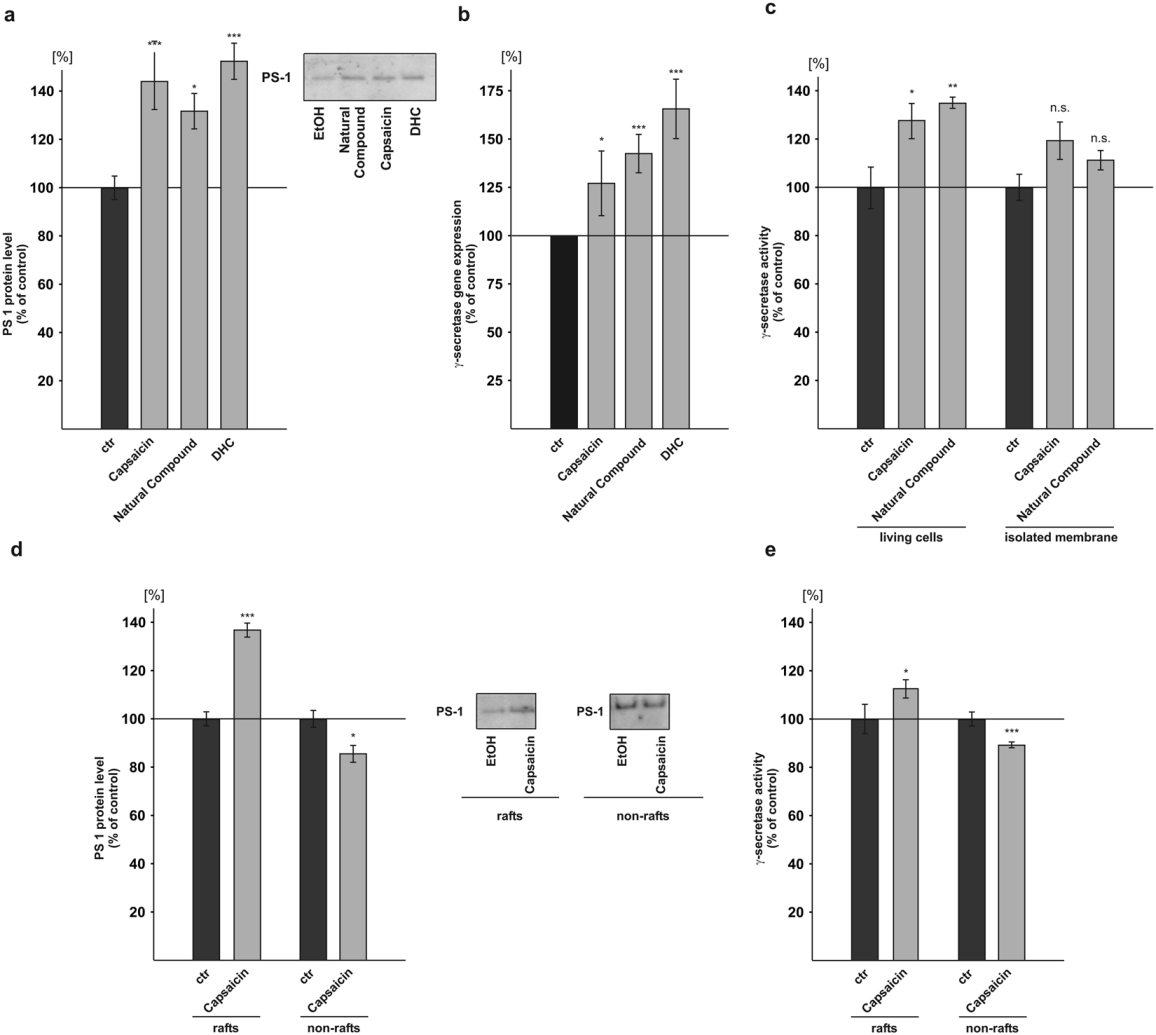
Effect of capsaicinoids on γ-secretase. (**a**) Levels of presenilin 1 (PS1) protein after treatment with capsaicin, natural compound and DHC on SH-SY5Y cells analyzed by WB (n ≥ 5). (**b**) Gene expression analysis of γ-secretase complex components (*APH1a, NCSTN, PSEN1, PSEN2, PSENEN*) in capsaicinoid treated SH-SY5Y cells (n = 4). (**c**) γ-secretase activity in living cells (n ≥ 9) and in isolated membranes (n = 3) of SH-SY5Y wt cells after capsaicin incubation. (**d**) Protein levels of PS1 in rafts and non-rafts after capsaicin incubation (n = 3). Representative, immunodetected PS1 signals are shown on the right. (**e**) γ-secretase activity in raft and non-raft fractions after capsaicin treatment (n = 3). Error bars represent the standard error of the mean. Asterisks show the statistical significance calculated by unpaired Student’s t test or ANOVA (*p ≤ 0.05; **p ≤ 0.01; ***p ≤ 0.001).

As we observed an increase in total Aβ levels in presence of capsaicinoids by the use of C99 expressing cells, we next analyzed PS1 protein levels. The PS1 protein levels were significantly increased to 144.3% in presence of capsaicin (capsaicin: 144.3% ± 11.9%, p = 0.001). Cells treated with the natural compound or DHC also revealed a significant increase in PS1 protein levels to 132.0% and 152.7%, respectively (natural compound: 132.0% ± 7.3%, p = 0.024; DHC: 152.7% ± 7.5%, p ≤ 0.001) (Fig. 4a). In line with elevated PS1 protein levels, capsaicin, the natural compound as well as DHC also significantly increased *PSEN1* mRNA expression as determined by RT-PCR (Table 1). The averaged expression of all γ-secretase components revealed a significant increase to 127.2% for capsaicin, to 142.5% for the natural compound and to 165.7% for DHC (capsaicin: 127.2% ± 13.4%, p = 0.050; natural compound: 142.5% ± 7.9%, p ≤ 0.001; DHC: 165.7% ± 12.3%, p ≤ 0.001) (Fig. 4b). The RT-PCR results for the single γ-secretase components including *APH1a*, *PSEN1* (PS1), *PSEN2* (PS2), *PSENEN* (presenilin enhancer) and *NCSTN* (nicastrin) are shown in Table 1. Similar to the determination of β-secretase activity, we examined γ-secretase activity in living cells as well as in purified membranes. Capsaicin significantly increased γ-secretase activity in living cells to 128.0% (capsaicin: 128.0% ± 7.3%, p = 0.026). Living cells incubated with the natural compound revealed a significant increase in γ-secretase activity to 135.2% (natural compound: 135.2% ± 2.3%, p = 0.007) (Fig. 4c). The determination of γ-secretase activity in living HEK as well as N2a cells in presence of the natural compound also showed an increase to 117.5% and 142.3% respectively, resulting in a mean γ-secretase activity elevation for all analyzed cell lines to 131.7% (±7.4%, p = 0.013) (see Supplemental Fig. S5). Similar to the effect of capsaicinoids on β-secretase activity in isolated membranes, we observed no significant direct effect of capsaicin and the natural compound on γ-secretase enzyme activity (Fig. 4c). Capsaicin and the natural compound only trended to elevate γ-secretase activity directly. As amyloidogenic APP processing has been shown to take place in lipid raft microdomains^76–78^, we analyzed γ-secretase processing in raft and non-raft microdomains. To determine raft localization of PS1, neuroblastoma cells incubated with capsaicin or solvent control were solubilized in Triton X-100 and separated by buoyant density centrifugation. Raft and non-raft membrane microdomains were identified by immunoblotting with flotillin, a raft marker protein, and cadherin for non-raft fractions (Supplemental Fig. S3). Flotillin-positive fractions were pooled and analyzed for PS1 protein levels. The PS1 protein levels  were significantly increased to 137.1% in lipid raft microdomains, whereas the PS1 protein levels  were reduced to 85.7% in pooled non-raft cadherin-positive fractions (PS1 protein rafts: 137.1% ± 2.9%, p ≤ 0.001; PS1 protein non-rafts: 85.7% ± 3.5%, p = 0.044) (Fig. 4d), indicating a shift of PS1 from non-raft to raft membrane microdomains in presence of capsaicin. The determination of γ-secretase activity in raft fractions revealed a statistically significant increase to 112.8% in presence of capsaicin, whereas γ-secretase activity was slightly but significantly decreased to 89.4% in non-raft fractions (γ-secretase activity rafts: 112.8% ± 3.8%, p = 0.034; γ-secretase activity non-rafts: 89.4% ± 1.2%, p ≤ 0.001) (Fig. 4e).

**Table 1:** mRNA levels of γ-secretase components.

	APH1a		NCSTN		PSEN1		PSEN2		PSENEN	
capsaicin	128 ± 67%	0.6077	132 ± 13%	0.0137*	128 ± 5%	0.0002***	137 ± 27%	0.1299	111 ± 11%	0.2637
natural compound	156 ± 19%	0.0055**	145 ± 18%	0.0119*	155 ± 19%	0.0073 **	153 ± 17%	0.0043 **	103 ± 3%	0.2783
dihydrocapsaicin	215 ± 30%	0.0012**	190 ± 18%	0.0002***	169 ± 23%	0.0048 **	156 ± 21%	0.0096 **	100 ± 14%	0.9905

RT-PCR results for the single γ-secretase components including *APH1a*, *NCSTN* (nicastrin), *PSEN1* (PS1), *PSEN2* (PS2) and *PSENEN* (presenilin enhancer) in human neuroblastoma cells after incubation of capsaicinoids. Mean ± standard error of the mean are listed on the left row and statistical significance calculated by unpaired Student’s t test or ANOVA (*p ≤ 0.05; **p ≤ 0.01; ***p ≤ 0.001) on the right row, respectively.

### Influence of capsaicinoids on cholesterol levels and cation channels

**Figure 5:**
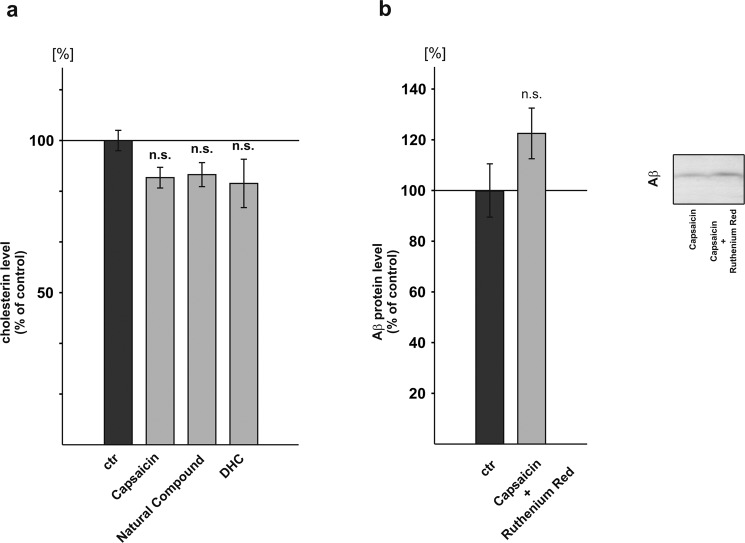
Influence of capsaicinoids on cholesterol levels and cation channels. (**a**) Cholesterol levels after capsaicinoid incubation in SH-SY5Y APP^695^ cells (n ≥ 3). (**b**) Effect of Ruthenium Red and capsaicin on Aβ protein levels in SH-SY5Y APP^695^ cells (n = 3). Error bars represent the standard error of the mean. Asterisks show the statistical significance calculated by unpaired Student’s t test or ANOVA (*p ≤ 0.05; **p ≤ 0.01; ***p ≤ 0.001).

High cholesterol levels are implicated in the pathogenesis of AD^79,80^ and capsaicin has been shown to reduce cholesterol^81^. We therefore analyzed whether capsaicin, the natural compound as well as DHC decrease cholesterol levels in our cell culture model. All analyzed compounds had a tendency to decrease cholesterol levels, however statistical analysis did not reach significance (capsaicin: 87.8% ± 1.0%, p = 0.196; natural compound: 88.8% ± 2.4%, p = 0.280; DHC: 85.9% ± 2.4%, p = 0.195) (Fig. 5a). However, one has to consider that in our experimental setting capsaicinoids were incubated for a short term (24 hours) and the effect of capsaicinoids on cholesterol might be more pronounced in the case of long-term incubation.

To investigate whether the observed effects of capsaicin on Aβ homeostasis are dependent on Ca^2+^ influx through cation channels, we incubated SH-SY5Y cells stably expressing APP^695^ in presence of capsaicin and Ruthenium Red. Ruthenium Red blocks cell membrane-located cation channels activated by capsaicin and blocks voltage-sensitive Ca^2+^ channels. If capsaicin would exert its effect via calcium influx, one would expect that by blocking these channels, secreted Aβ levels should be unchanged or decreased in presence of capsaicin and Ruthenium Red compared to cells only treated with capsaicin. However, in presence of capsaicin and Ruthenium Red secreted Aβ levels were not significantly elevated to 122.8% (± 10.5%, p = 0.199) compared to control cells incubated with capsaicin (Fig. 5b), indicating that the effect of capsaicin on Aβ homeostasis is independent of calcium influx in presence of capsaicin, which is in line with the finding that SH-SY5Y cells do not express TRPV1 channels^82^ and capsaicin therefore did not induce a calcium influx (Supplemental Fig. S2a). As a control, transient expression of *TRPV1* in SH-SY5Y cells resulted in an increased basal as well as capsaicin induced calcium influx (Supplemental Fig. S2b).

### Capsaicinoids decrease Aβ degradation by affecting IDE

Besides Aβ anabolism, total Aβ levels strongly depend on Aβ catabolism by Aβ degrading enzymes^18,19^. To examine Aβ degradation in presence of capsaicinoids we incubated mouse neuroblastoma cells (N2a) with capsaicin, the natural compound or DHC in presence of human synthetic Aβ peptides. Aβ degradation was monitored by immunoblotting with antibody W02 recognizing human but not endogenous mouse Aβ. By utilizing the W02 antibody and adding Aβ peptides with the human Aβ sequence to a mouse cell line with neuronal properties, it is possible to measure the Aβ degradation independently of the endogenous Aβ production (see Supplemental Fig. S6).

**Figure 6:**
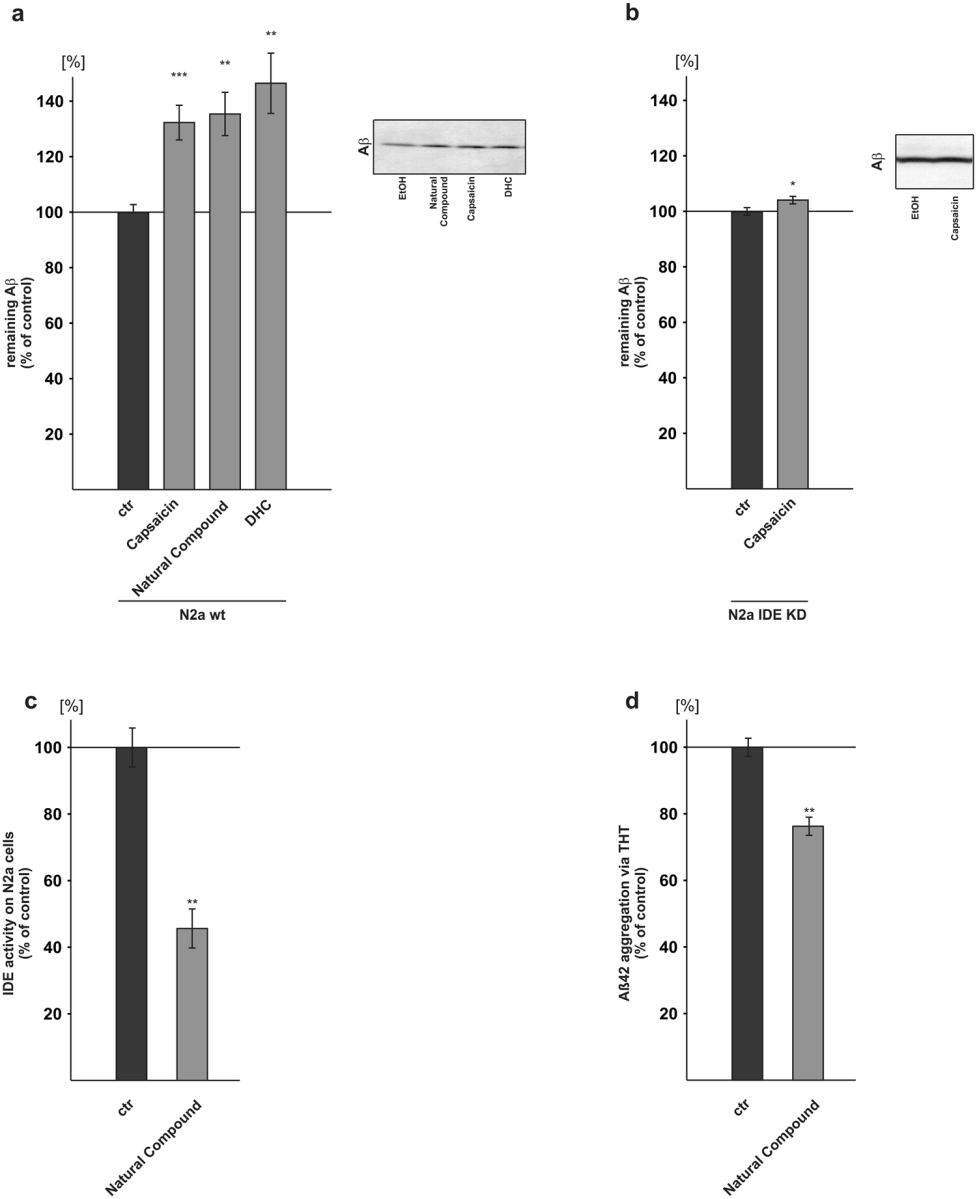
Effect of capsaicinoids on Aβ degradation and aggregation. (**a**) Aβ degradation was examined in capsaicinoid treated murine neuroblastoma cells (N2a) by WB (n ≥ 9). (**b**) Influence of capsaicin on degradation of Aβ in N2a IDE knock-down cells (n = 9). (**c**) IDE activity assay in N2a cells after treatment with natural compound (n = 6). (**d**) THT-based Aβ aggregation assay (n = 9). Error bars represent the standard error of the mean. Asterisks show the statistical significance calculated by unpaired Student’s t test or ANOVA (*p ≤ 0.05; **p ≤ 0.01; ***p ≤ 0.001).

In presence of capsaicin, remaining human synthetic Aβ peptides were significantly increased to 132.6%, indicating impaired Aβ catabolism. Similarly, for the other capsaicinoids, remaining Aβ peptides were significantly elevated to 135.7% in presence of the natural compound. An even stronger effect was observed for DHC revealing an increase to 146.8% (capsaicin: 132.6% ± 3.6%, p ≤ 0.001; natural compound: 135.7% ± 6.2%, p ≤ 0.002; DHC: 146.8% ± 8.4%, p = 0.002) (Fig. 6a). Aβ degradation in neuronal cell lines is mainly dependent on IDE and neprilysin^83^. To further investigate whether the observed impaired Aβ degradation upon capsaicinoid treatment is mediated by IDE, we generated N2a cells knocked-down for IDE (see Supplemental Fig. S4), one of the main enzymes involved in Aβ degradation. Capsaicin only slightly increased remaining human Aβ peptides to 104.25% (capsaicin: 104.25% ± 1.3%, p = 0.038) in IDE knock-down cells (Fig. 6b) compared to the observed increase to 132.6% in wild type N2a, indicating that IDE is affected by capsaicin. To confirm these results, an IDE activity assay was performed in presence of capsaicinoids revealing also a decreased IDE activity to 45.6% (±11.7%, p = 0.008) (Fig. 6c).

### Effect of capsaicinoids on Aβ aggregation

Besides Aβ anabolism and Aβ catabolism, aggregation of Aβ peptides to small oligomers plays an important role in AD pathogenesis^84^. Therefore, we monitored Aβ aggregation by the use of a fluorescent Thioflavin T-based assay. In presence of capsaicinoids (natural compound) Aβ aggregation was significantly decreased to 76.3% (±5.4%, p = 0.010) (Fig. 6d).

## Discussion

AD is a multifactorial disease and its pathogenesis involves multiple pathological processes. According to the amyloid cascade hypothesis, Aβ accumulation in the brain of affected individuals causes synaptic dysfunction and neuronal cell death, leading to the well-characterized symptoms of the disease^85,86^. AD has a long preclinical stage and Aβ generation starts years or decades before first symptoms appear to unambiguously manifest the disease. One of the current scientific approaches in respect to AD is to potentially delay the disease onset by bioactive compounds lowering Aβ generation or Aβ aggregation. Several bioactive and/or dietary compounds have been described, which might be beneficial to treat or prevent AD, the most investigated being DHA^32,87–89^. Curcumin, a natural polyphenol of *Curcuma* species, has a structure similar to capsaicin and is discussed to reduce Aβ levels and Aβ aggregation^90–92^. Beside Aβ aggregation, oxidative stress and inflammation have been described as important factors to cause AD^93,94^.

**Figure 7:**
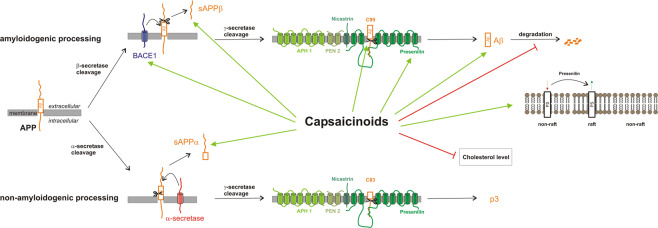
Mechanism of capsaicinoids action.

Capsaicin, the most prominent vanilloid in the fruits of *Capsicum* species, exerts pleiotropic pharmacological activities, including anti-inflammatory and anti-oxidant functions, which might be beneficial in respect to AD. However, so far the molecular mechanism how capsaicin affects APP processing and thus the generation of toxic Aβ species is not understood. In the present study we elucidated the effect of capsaicin, DHC and the natural compound consisting of several capsaicinoids on APP processing. All analyzed capsaicinoids significantly increased Aβ generation in the neuroblastoma cell line SH-SY5Y stably transfected with APP^695^ or the truncated construct C99, which only requires cleavage by γ-secretase to release Aβ. Elevated Aβ generation in presence of capsaicinoids was verified in the non-neuronal cell line COS7. The capsaicinoid induced elevation in total secreted Aβ levels are caused by an increase in gene transcription of β-secretase *BACE1* and the components of the γ-secretase complex, leading to elevated protein levels of BACE1 and PS1, the catalytically active component of the γ-secretase complex^12,13^. Additionally, in presence of capsaicinoids PS1 shifts from non-raft to raft membrane microdomains, discussed to be involved in amyloidogenic APP processing^95^. The observed elevation in gene transcription of the amyloidogenic secretases resulted in an increased enzyme activity of β- and γ-secretase detected in living SH-SY5Y cells incubated with capsaicinoids. Therefore, capsaicinoids exert their Aβ increasing effect rather indirectly, further substantiated by our finding that by the use of purified membranes no or only a marginal direct effect of capsaicinoids on β- or γ-secretase activity was observed. The increase of β- and γ-secretase activity was further confirmed in living HEK and N2a cells. No significant differences between capsaicin, dihydrocapsaicin and the natural compound were found in respect to Aβ generation, and the effect on the Aβ release seems to be independent of the single capsaicinoid structure. Besides increasing amyloidogenic APP processing, capsaicinoids decrease Aβ degradation by affecting IDE, also contributing to an elevation in total Aβ levels (Fig. 7). Our findings are in line with a previous study reporting that capsaicin promotes the amyloidogenic route of brain APP processing in capsaicin-treated rats^41^. In contrast, a recent study proposes that a capsaicin-rich diet may have protective effects on AD patients. In that study, a capsaicin-rich diet in humans, whose dietary habits regarding chili pepper consumption were collected using a Food Frequency Questionnaire, reduced serum Aβ40 levels as well as total serum Aβ levels, and revealed positive effects on cognitive functions. Serum Aβ42 levels were unaffected^40^. However, as discussed by the authors, blood Aβ has limitations as a marker to reflect AD related pathological changes. The analysis of cerebrospinal fluid would be more favorable. Nevertheless, further studies addressing cognitive impairment in mice proposed beneficial effects of capsaicin in mice. Chen *et al*. reported that daily treatment with capsaicin (1 mg/kg, i.p.) improved spatial learning as well as memory synaptic function in C57Bl/6 mice microinjected with 100 µM Aβ42^96^. Treatment with the TRPV1 antagonist capsazepine (1 mg/kg, i.p.) showed no effects on cognitive and synaptic function in these mice, suggesting that the above mentioned effect may be mediated through the activity of TRPV1 channels. Similarly, capsaicin has been shown to ameliorate cognitive impairment and tau hyperphosphorylation provoked by cold water stress in rats^39^. Also, reduced hyperphosphorylation of tau proteins has been reported in the hippocampus of type2 diabetes rat, known as risk factor for the development of AD, fed with a capsaicin-rich diet^37^. Interestingly, the capsaicin diet did not reduce tau hyperphosphorylation in healthy rats not affected by type2 diabetes, indicating that dietary capsaicin might prevent AD in individuals affected by type2 diabetes. Capsaicin, present as bioactive compound in Kimchi, a traditional Korean fermented vegetable, also had a tendency to decrease phosphorylated tau proteins in the brain of Aβ-injected mice^97^. In this study protein expression of APP and BACE1, elevated by Aβ injection, also tended to decrease by oral administration of 10 mg capsaicin. Furthermore, daily oral administration of bioactive compounds in Kimchi, including capsaicin (10 mg/kg), improved cognitive and memory functions in mice injected with Aβ25-35^98^. Recently it has been shown that TRPV1 activation by capsaicin rescued Aβ-induced degradation of hippocampal gamma oscillations, a cognition-relevant EEG pattern, by reversing desynchronization of AP firing in CA3 pyramidal cells and by reversing the shift in the excitatory/inhibitory current balance^99^. This reversing effect was not detectable in TRPV1 knock-out mice or in presence of the antagonist capsazepine, clearly pointing towards a role of TRPV in mediating these effects.

Although these studies propose beneficial effects in tests of cognition and show decreased tau hyperphosphorylation associated with AD, our results indicate that capsaicinoids as bioactive compounds have additional effects on APP processing and Aβ homeostasis. In the present study capsaicinoids significantly increased total Aβ levels by increasing amyloidogenic APP processing and by reducing Aβ degradation and might therefore not be unconditionally recommended as a dietary supplement to prevent AD or to delay the disease onset. On the other hand we also found beneficial effects of capsaicinoids in respect to AD as we could show that capsaicinoids reduced Aβ aggregation. Interestingly, vanillin and curcumin which are structurally related to capsaicinoids have been shown to decrease Aβ aggregation^100^. Our results imply that potentially a TRPV1 agonist that does not affect APP processing might be preferential, which has to be proven in further studies.

Given the multifactorial nature of the disease including various pathological processes, it is difficult to judge whether a compound might be beneficial or detrimental when only one pathological aspect in AD, e.g. Aβ generation or tau hyperphosphorylation, is investigated.

As a limitation of our study, it has to be pointed out that this is a sole *in vitro* study aiming to elucidate the molecular effect of capsaicinoids on Aβ homeostasis. Our study investigates the effect of capsaicinoids on SH-SY5Y cells in a short time period and in the absence of functional TRPV1 channels. The concentration used in this study is supraphysiological. It has to be highlighted that capsaicinoids are one of the most frequently consumed spices in humans. A (life)-long exposure of a continuous but low concentration is hard to simulate in cell culture. Therefore, we decided to use 24 hours long exposure of a higher concentration to investigate if there is in principle an impact of capsaicinoids on Aβ homeostasis or the amyloidogenic pathway. The rationale for using 10 µM capsaicinoids was that at this concentration no cytotoxicity was observed and other studies have used a similar or higher concentration range to determine the potential effect of capsaicinoids on different cellular pathways^101–106^.

Moreover, dietary habits such as persistent consumption of spicy food might therefore have long term effects, potentially mediated by TRPV1, which were not reflected in this study. E.g. one might assume that the cholesterol-lowering effect would be more pronounced *in vivo*, when capsaicin is consumed over a long period by diet.

As the experiments were performed in SH-SY5Y cells and as these cells do not express TRPV1^82^, which was experimentally verified by us, our results show that the observed increase in Aβ levels is likely independent of a change in calcium homeostasis. However, we do not rule out that in other cells highly expressing the TRPV1 channels, potential changes in calcium homeostasis induced by capsaicin might also interfere with the Aβ homeostasis. However, besides increasing total Aβ levels, in our study capsaicinoids revealed also an increase in sAPPα levels. The sAPPα fragment has neurotrophic and neuroprotective functions and is reported to be decreased in some cases of AD^107,108^.

In summary, capsaicin has been shown in literature to be beneficial in respect to tau pathology in AD and by reducing ROS and inflammation. Here we show additional and potential undesirable effects of capsaicin on APP processing and Aβ homeostasis, emphasizing the need for further studies before a clear advice of capsaicin in respect to AD prevention and treatment can be given.

## Supplementary information

Supplementary information.

## Data Availability

All data generated or analysed during this study are included in this published article (and its Supplementary Information files).
