# Supplementary material for: The impact of capsaicinoids on APP processing in Alzheimer’s disease in SH-SY5Y cells
Source: Sci Rep. 2020 Jun 8;10:9164. doi: 10.1038/s41598-020-66009-6 (PMC7280252; doi:10.1038/s41598-020-66009-6)
Supplement: Supplementary file 1 — Supplementary information. [file 41598_2020_66009_MOESM1_ESM.docx]

**Supplementary Information**

**The impact of capsaicinoids on APP processing in Alzheimer’s disease in SH-SY5Y cells**

Marcus O.W. Grimm ^1,2, ŧ, *^, Tamara Blümel ^1, ŧ^, Anna A. Lauer ^1^, Daniel Janitschke ^1^, Christoph Stahlmann ^1^, Janine Mett ^1,3^, Viola J. Haupenthal ^1^, Anna-Maria Miederer ^4^, Barbara A. Niemeyer ^4^, Heike S. Grimm ^1^, Tobias Hartmann ^1,2,5^

^1^ Experimental Neurology, Saarland University, Homburg / Saar, Germany

^2^ Neurodegeneration and Neurobiology, Saarland University, Homburg / Saar, Germany

^3^ Biosciences Zoology / Physiology-Neurobiology, Faculty NT – Natural Science and Technology, Saarland University, Saarbrücken, Germany

^4^ Molecular Biophysics CIPMM, Saarland University, Homburg / Saar, Germany

^5^ Deutsches Institut für DemenzPrävention (DIDP), Saarland University, Homburg / Saar, Germany

***** Correspondence: [marcus.grimm@mx.uni-saarland.de](mailto:marcus.grimm@mx.uni-saarland.de)

^ŧ^ these authors contributed equally to this work


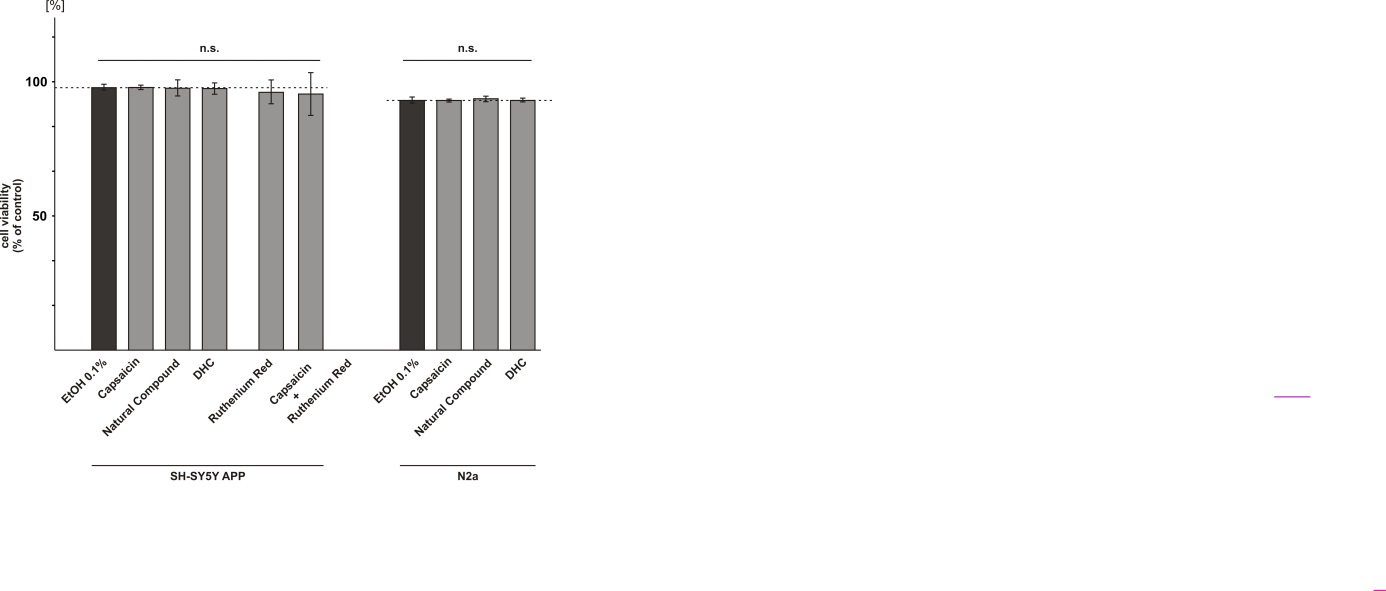


**Supplemental Figure S1: Influence of capsaicinoid incubation on cytotoxicity.** Levels of cytotoxicity were examined by LDH-Assay in SH-SY5Y APP^695^ and N2a cells after incubation of ethanol as solvent control, capsaicin, natural compound and DHC. Error bars represent the standard error of the mean. Asterisks show the statistical significance calculated by unpaired Student’s t test or ANOVA (* p ≤ 0.05; ** p ≤ 0.01; *** p ≤ 0.001).


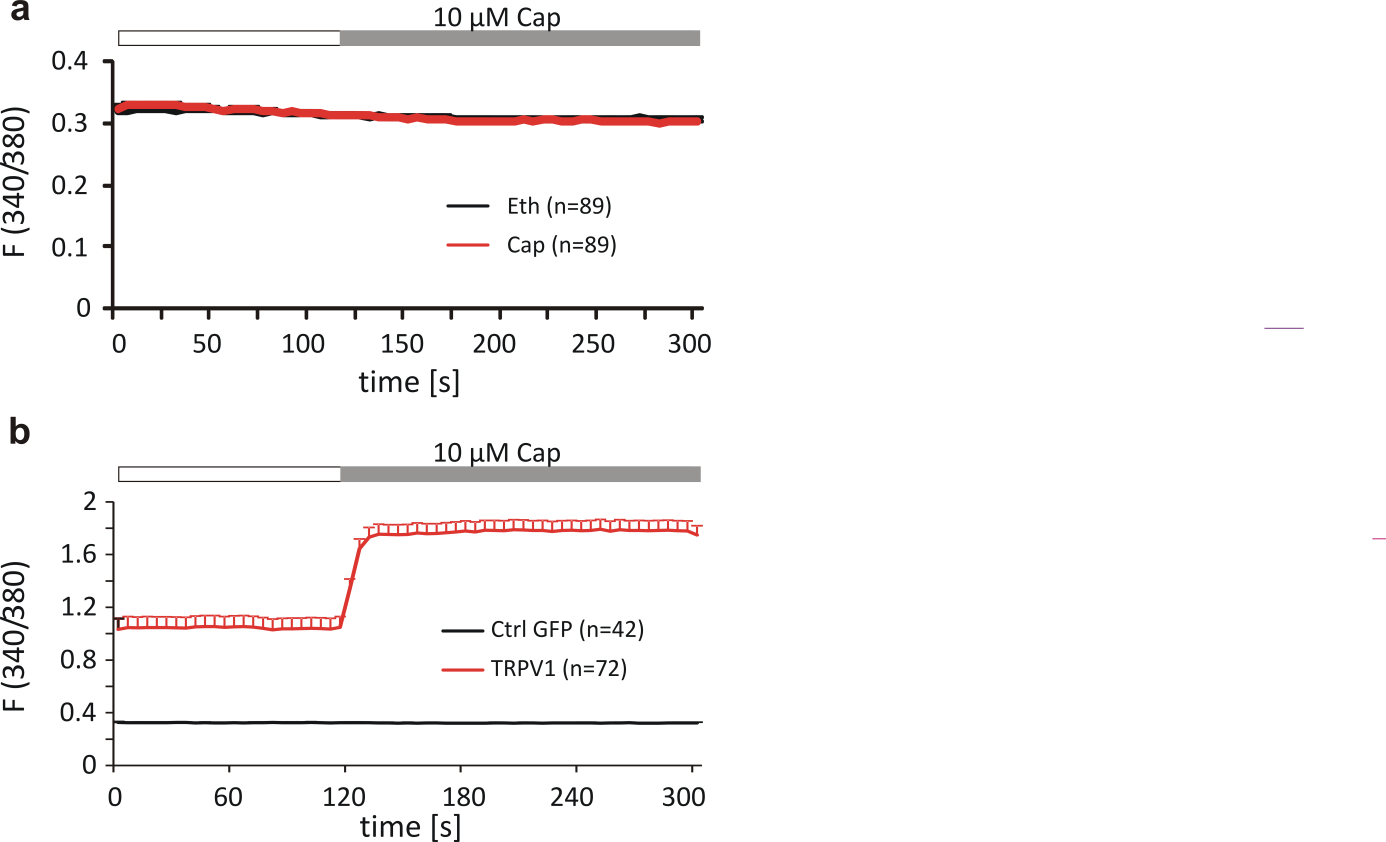


**Supplemental Figure S2:** **Influence of capsaicin on Ca^2+^ homeostasis.** (a) Fura 2 ratios before and after addition of 10 µM capsaicin to SH-SY5Y APP^695^ cells. (b) Effect of capsaicin on SH-SY5Y APP^695^ cells transfected 24 h prior to measurement with either *TRPV1* or Ctrl *GFP* vectors. n = 3 independent measurements; n = # of cells, see figure. Error bars represent the standard error of the mean.


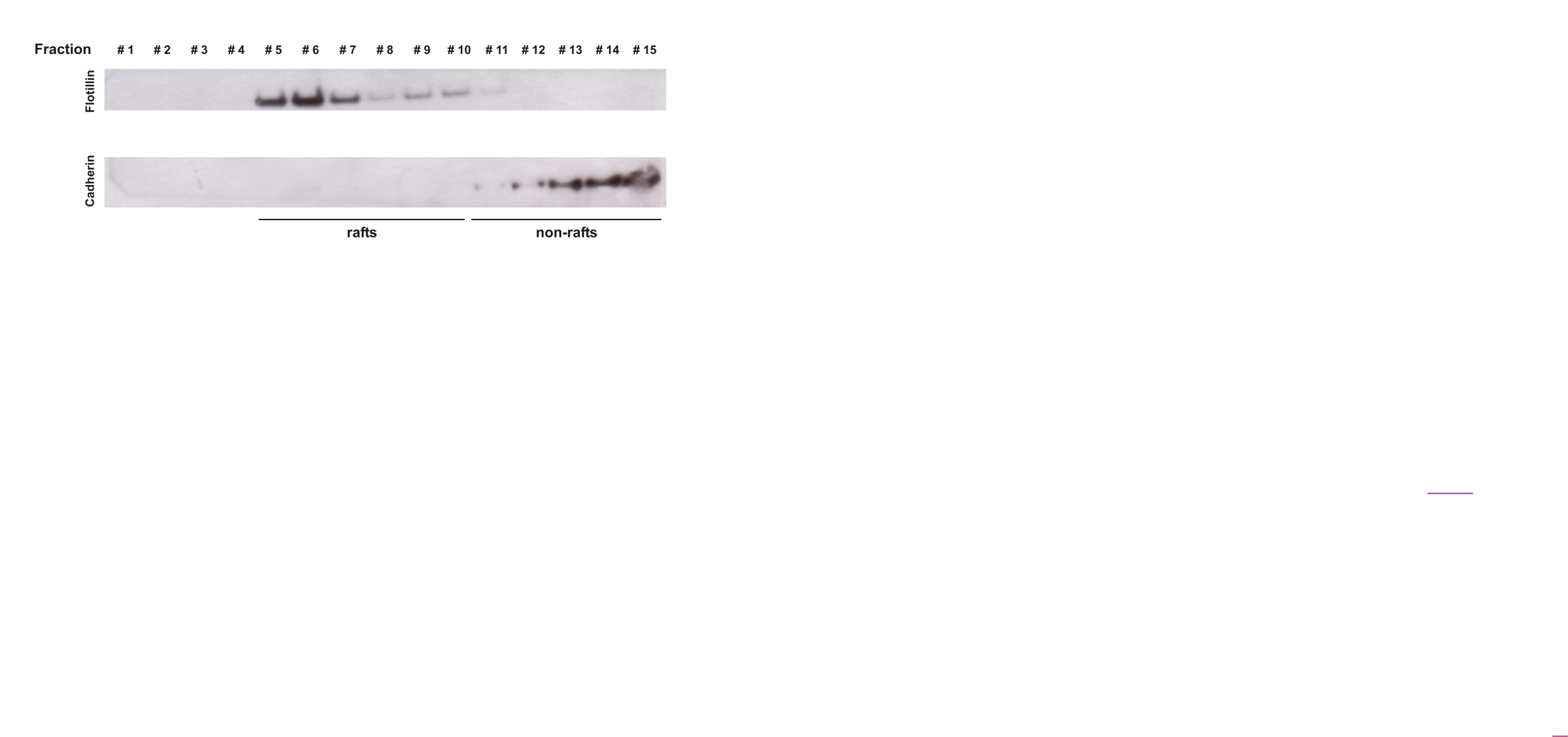


**Supplemental Figure S3:** **Preparation of lipid rafts.** Flotillin and cadherin WB analysis.


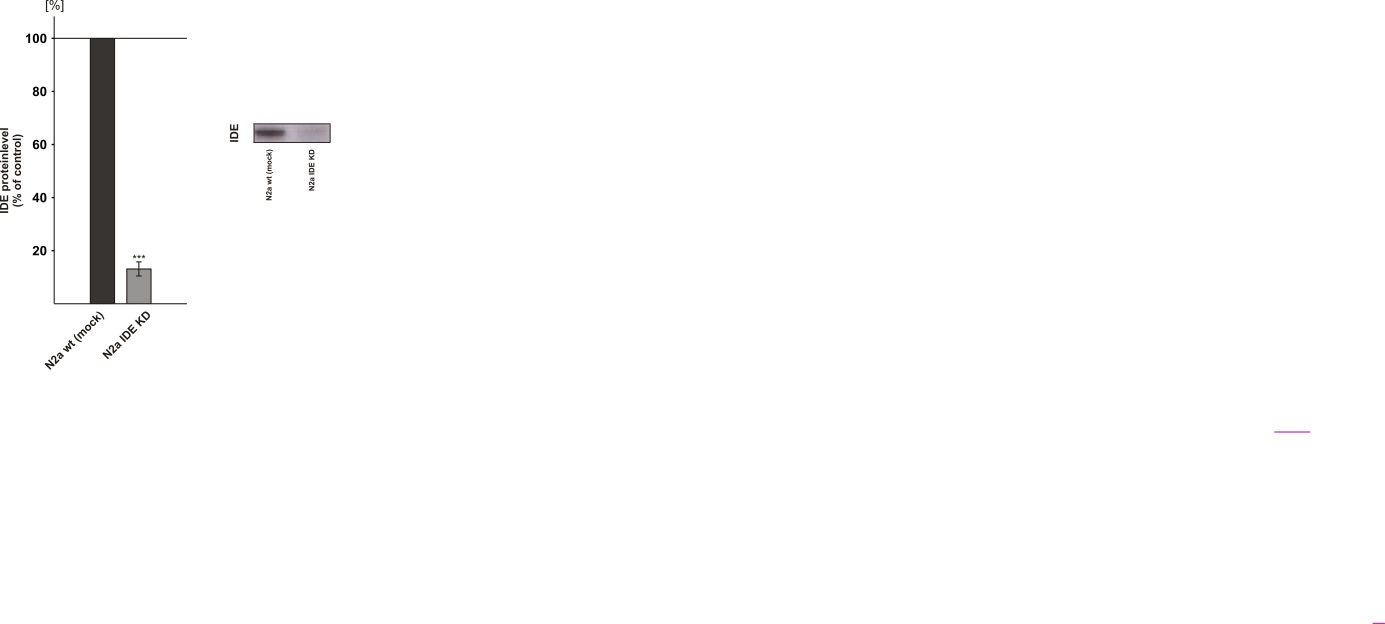


**Supplemental Figure S4: IDE knock-down efficiency in N2a cells.** Expression of *IDE* in N2a cells after shRNA mediated knock-down. Error bars represent the standard error of the mean. Asterisks show the statistical significance calculated by unpaired Student’s t test (* p ≤ 0.05; ** p ≤ 0.01; *** p ≤ 0.001).

**
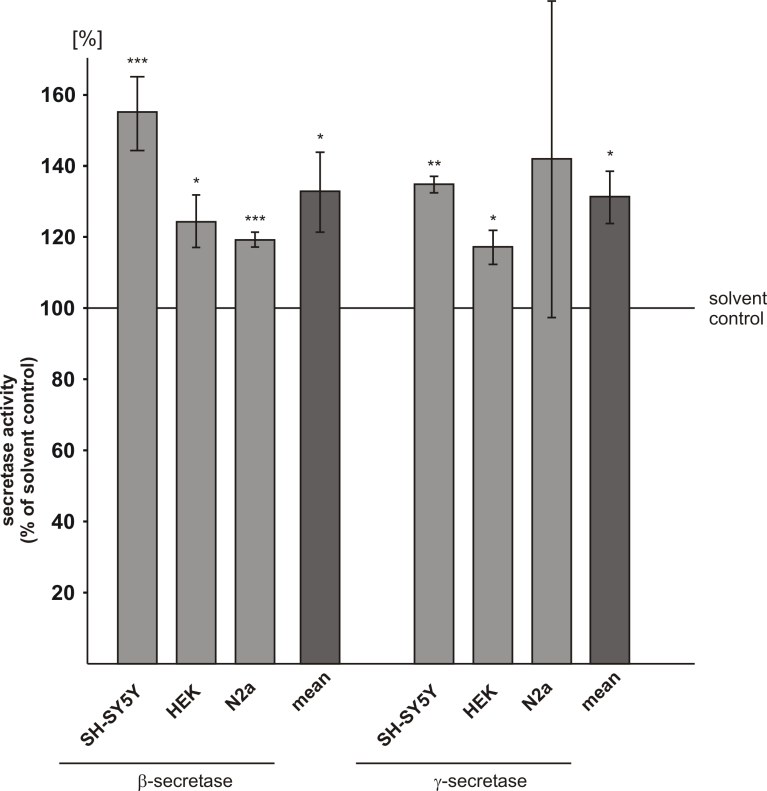
**

**Supplemental Figure S5:** β- and γ-secretase activity in SH-SY5Y, HEK and N2a in living cells. Error bars represent the standard error of the mean. Asterisks show the statistical significance calculated by unpaired Student’s t test (* p ≤ 0.05; ** p ≤ 0.01; *** p ≤ 0.001).


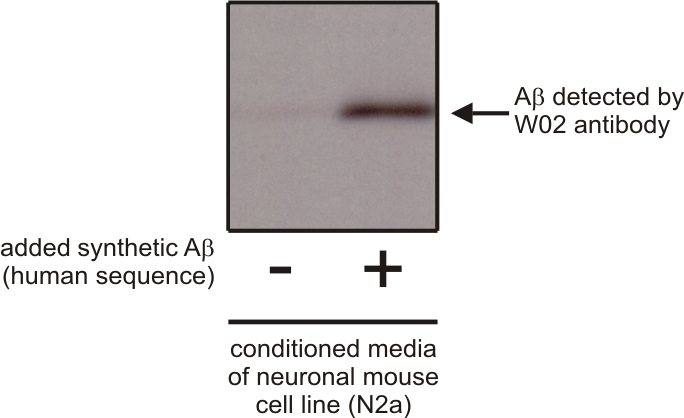


**Supplemental Figure S6:** Control experiment of Aβ degradation. W02 antibody mediated Aβ detection of conditioned media of N2a cells in absence (-) and presence (+) of supplemented synthetic Aβ with human sequence. As the W02 antibody detects only supplemented human Aβ, the Aβ detection in figure 6a, utilizing the same W02 antibody, is only dependent on Aβ degradation and independent of endogenous Aβ production of N2a cells.

**
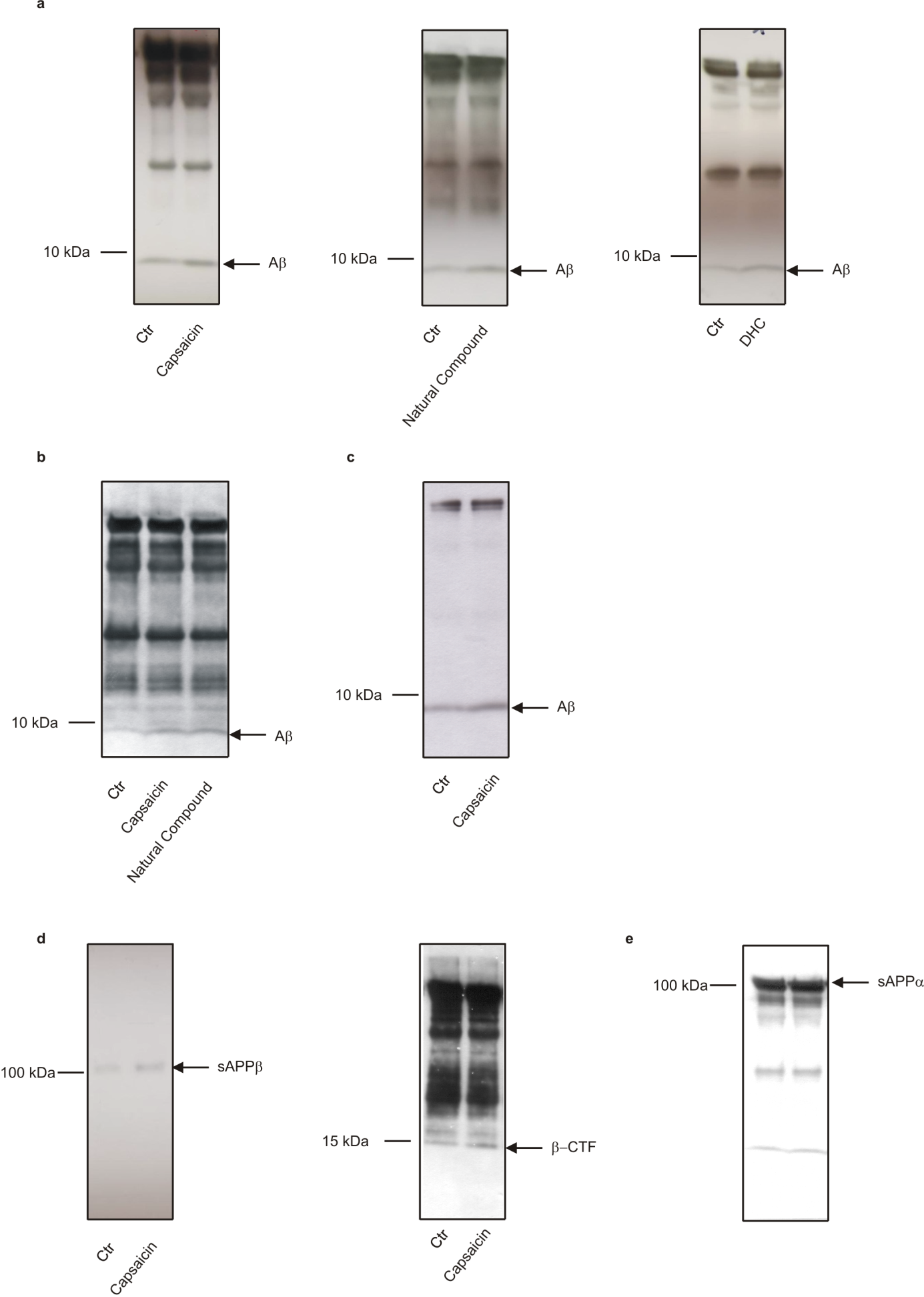
**

**
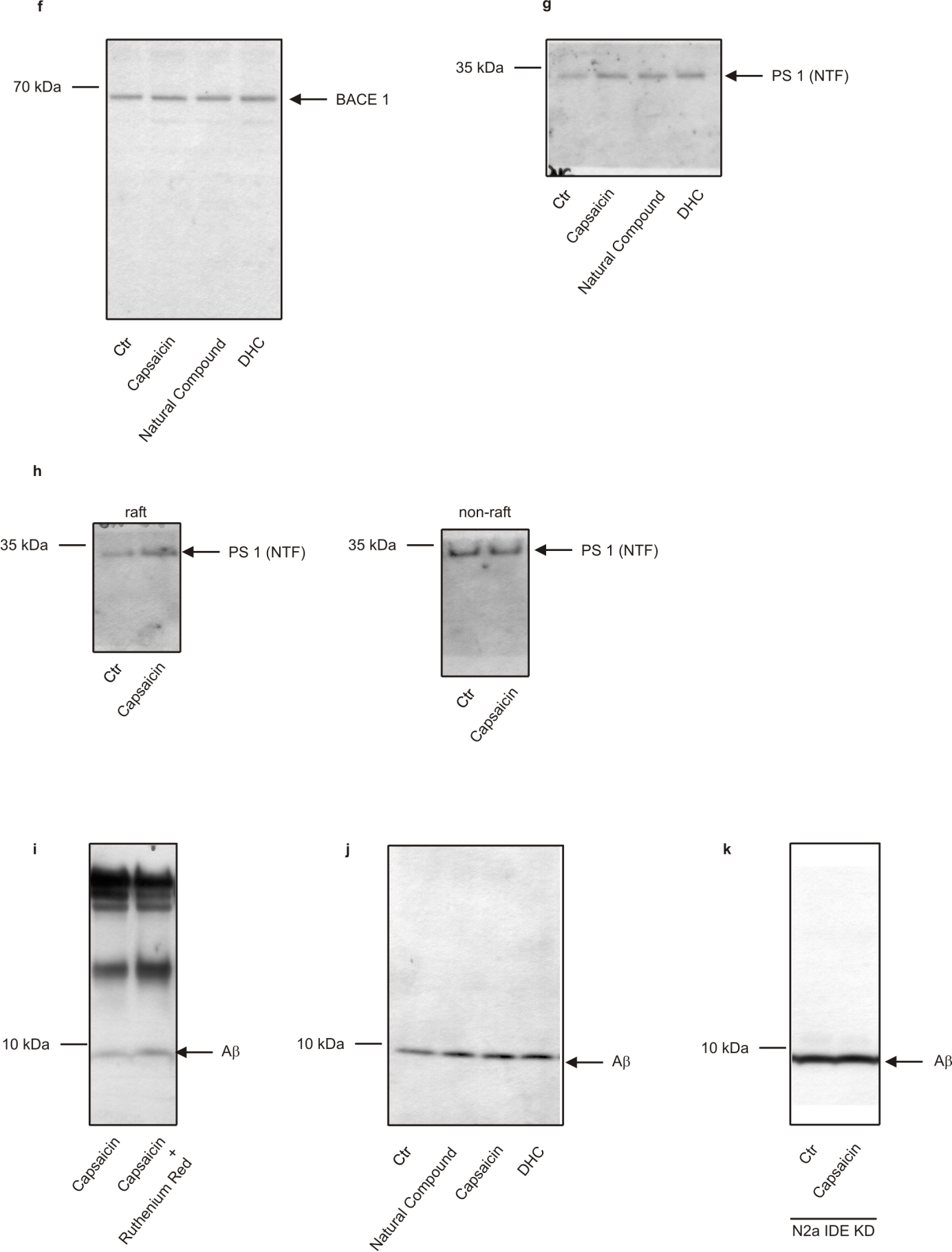
**

**
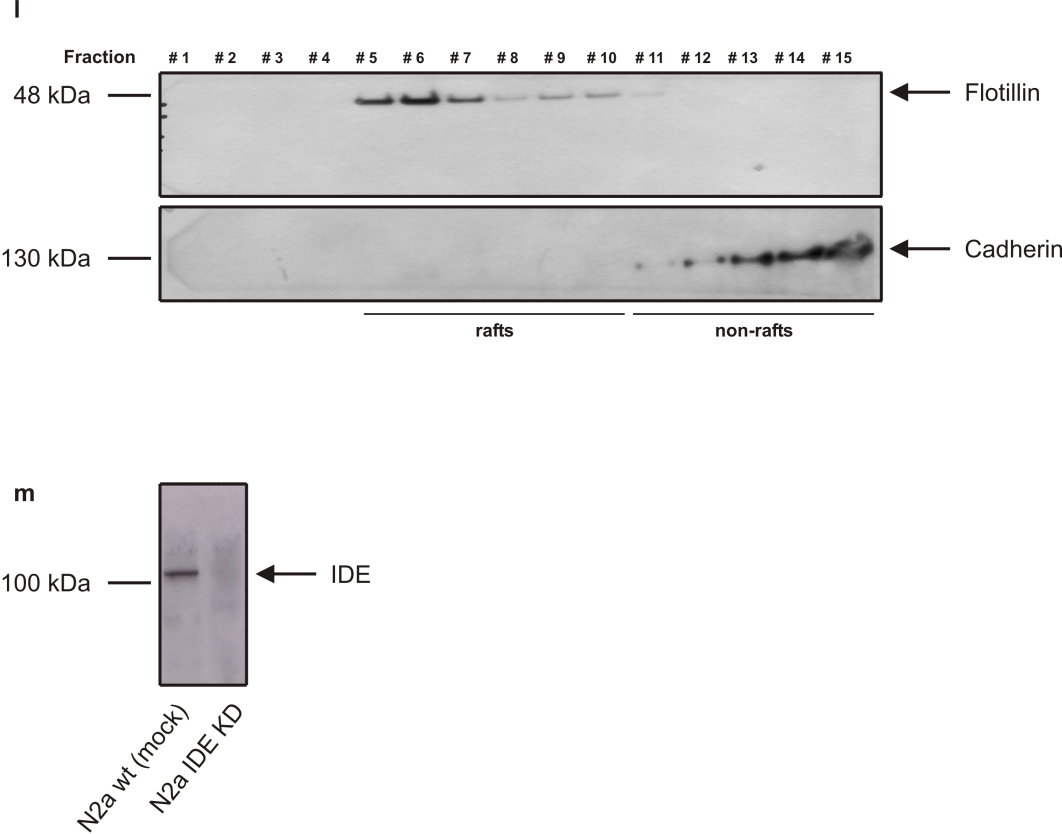
**

**Supplemental Figure S7:** In accordance to journal guidelines uncropped Western blots, which were presented in the main figures. (a) Western blot / immunoprecipitation (IP) shown in figure 2a. (b) Western blot / IP shown in figure 2b. (c) Western blot / direct load shown in figure 2c. (d) Western blot / direct load (sAPPβ) and Western blot / IP (β-CTF) shown in figure 2d. (e) Western blot / IP shown in figure 2e. (f) Western blot / direct load shown in figure 3a. (g) Western blot / direct load shown in figure 4a. (h) Western blot / direct load shown in figure 4d. (i) Western blot / direct load shown in figure 5b. (j) Western blot / direct load shown in figure 6a. (k) Western blot / direct load shown in figure 6b. (l) Western blot shown in figure S3. The Western blot membrane was cut in order to save antibody. The complete membrane, which was incubated with the first antibody (flotillin or cadherin) is presented. (m) Western blot shown in figure S4.

**Detailed description of the used methods.**

1. Preparation of Isolated Membranes

After the postnuclear fractions were pre-warmed and incubated with 10 µM of capsaicinoids for 20 minutes at 37 °C, the samples were centrifuged for pelleting the membranes at 55.000 rpm for 75 min and 4 °C. The supernatant was discarded and the membrane pellets were homogenized with Minilys (Peqlab, Erlangen, Germany) using glass beads. Afterwards samples were resuspended using 10 strokes with a cannula with a diameter of 0.4 mm, before secretase activity was measured.

1. Lipid Raft Preparation

For the preparation of lipid rafts, cells were homogenized in MES-buffered saline containing 0.1 % Triton X-100 (25 mM MES, pH 6.5; 150 mM NaCl) and protein amount was adjusted to 3 mg/ml. Three sucrose layers were added to 1.2 ml of homogenate one after the other: 1.2 ml MBS, 90 % sucrose, 0.1 % Triton X-100 (1); 4 ml MBS, 35 % sucrose, 0.1 % Triton X-100 (2); 4.5 ml MBS, 5 % sucrose, 0.1 % Triton X-100. Afterwards, buoyant density centrifugation at 35,000 rpm and 4 °C was performed for 18 hours in a SW40 rotor of a Beckman ultracentrifuge. Fractions of 650 µl were collected and the presence of the lipid raft marker flotillin and as well as of cadherin was examined by western blot (WB) analysis using the antibodies 610821 from BD Biosciences (Anti-Flotillin-1, 1:250) and ab6528 from Abcam (Anti-pan Cadherin, 1:1000). Activity of γ-secretase was analyzed directly from the fractions.

1. Immunoprecipitation

For the immunoprecipitation of Aβ and β-CTF all growth media samples were adjusted to the same protein amount and 20 µl protein G-Sepharose (Sigma, Taufkirchen, Germany) and W02 antibody (10 µg/µl; Millipore, Billerica, MA, USA) were used. After overnight overhead shaking, the washed precipitates were separated in 10-20 % Tris-Tricine gels (anamed Elektrophorese GmbH, Rodau, Germany) and further used for WB experiments.

1. Secretase Activity Assays

For examining the activity of β- or γ-secretase, incubated SH-SY5Y wild type cells were washed twice with pre-warmed live cell imaging solution (140 mM NaCl, 5 mM KCl, 8 mM CaCl_2_, 1 mM MgCl_2_, 20 mM HEPES, pH 7.4). Secretase substrates were diluted in imaging solution (20 µM β-secretase substrate, Calbiochem, 565758; 6.25 µM γ-secretase substrate, Calbiochem, 565764) and 50 µl were added to each well. Fluorescence was measured using a Safire^2^ Fluorometer (Tecan, Crailsheim, Germany) at excitation wavelengths of 345 ± 5 nm (β) / 355 ± 10 nm (γ) and emission wavelengths of 500 ± 5 nm (β) / 440 ± 10 nm (γ). For determination of secretase activity in isolated membranes and raft fractions those samples were adjusted to the same protein amount and the appropriate substrate was added.
